# Mitochondrial biogenesis, telomere length and cellular senescence in Parkinson’s disease and Lewy body dementia

**DOI:** 10.1038/s41598-022-22400-z

**Published:** 2022-10-20

**Authors:** Muhammad Asghar, Amani Odeh, Ahmad Jouni Fattahi, Alexandra Edwards Henriksson, Aurelie Miglar, Shervin Khosousi, Per Svenningsson

**Affiliations:** 1 Department of Biology, Lund University, Lund, Sweden; 2 Division of Infectious Diseases, Department of Medicine Solna, Karolinska Institutet, Solna, Sweden; 3 Department of Medical Biochemistry and Microbiology, Uppsala University, Uppsala, Sweden; 4 Department of Clinical Neuroscience, Karolinska Institutet, Solna, Sweden; 5 Basal and Clinical Neuroscience, Institute of Psychiatry, King’s College London, Psychology & Neuroscience, London, UK

**Keywords:** Dementia, Parkinson's disease, Cellular neuroscience

## Abstract

Progressive age is the single major risk factor for neurodegenerative diseases. Cellular aging markers during Parkinson’s disease (PD) have been implicated in previous studies, however the majority of studies have investigated the association of individual cellular aging hallmarks with PD but not jointly. Here, we have studied the association of PD with three aging hallmarks (telomere attrition, mitochondrial dysfunction, and cellular senescence) in blood and the brain tissue. Our results show that PD patients had 20% lower mitochondrial DNA copies but 26% longer telomeres in blood compared to controls. Moreover, telomere length in blood was positively correlated with medication (Levodopa Equivalent Daily Dose, LEDD) and disease duration. Similar results were found in brain tissue, where patients with Parkinson’s disease (PD), Parkinson’s disease dementia (PDD) and Dementia with Lewy Bodies (DLB) showed (46–95%) depleted *mtDNA* copies, but (7–9%) longer telomeres compared to controls. In addition, patients had lower mitochondrial biogenesis (*PGC-1α and PGC-1β*) and higher load of a cellular senescence marker in postmortem prefrontal cortex tissue*,* with DLB showing the highest effect among the patient groups. Our results suggest that mitochondrial dysfunction (copy number and biogenesis) in blood might be a valuable marker to assess the risk of PD. However, further studies with larger sample size are needed to evaluate these findings.

## Introduction

Parkinson’s disease (PD) is the second most common age-related neurodegenerative disease, characterized by motor dysfunctions caused by the progressive death of dopaminergic neurons in the substantia nigra, and is often accompanied by non-motor symptoms such as dementia, mood and sleep disorders^1–3^. Although PD is a complex disease with several causes, including genetic and environmental factors, progressive age remains the single major risk factor for PD^3^.

Aging is characterized by a time-dependent progressive deterioration of an organism’s function, caused by the accumulation of deleterious changes throughout its lifetime^4^. Cellular aging markers such as mitochondrial dysfunction and telomere shortening have been associated with age related disorders and neurodegenerative diseases^5–7^. Mitochondria are surrounded by double-membranes, which maintain the functional and structural integrity of pre- and post-mitotic cells, through involvement in cellular bioenergetics and the production of reactive oxygen species (ROS)^8^. Lower blood mitochondrial DNA (*mtDNA*) copy number has been associated with high mortality, poor health conditions, worse physical performance, and cognitive impairment^9^. Somatic *mtDNA* damage and mutations are part of the natural aging process, however, they have also been linked to age associated diseases and neurodegeneration in humans^7,10–12^. Furthermore, increased accumulation of *mtDNA* mutations and damage has been shown to contribute to impaired mitochondrial respiration^12,13^. Hence, mitochondrial DNA content and function might represent a valuable biomarker to monitor early changes in different physiological and pathological states.

Telomere shortening is a well-studied hallmark of aging that has been associated with several age-related disorders, infectious diseases and neurodegenerative diseases^14–17^. Telomeres are non-coding, ribonucleotide structures composed of highly conserved repetitive hexamer 5′-TTAGGG-3′ and a core of proteins called shelterin. Telomeres maintain chromosome integrity by capping the ends to prevent end-to-end joining of chromosomes and prevent loss of coding DNA sequences during DNA replication. Telomeres shorten progressively over time until reaching a critical length that leads to cell-cycle arrest, senescence, or apoptosis, respectively^4,18^. Whether telomere shortening also contributes to the pathogenesis of neurodegenerative disorders remains to be understood. Previous studies provide inconclusive findings regarding the association between telomere length and PD, where both shorter and longer telomeres have been identified as a risk factor for PD^17,19^.

Furthermore, it has been shown that shorter telomeres and dysfunctional mitochondria in turn lead to cellular senescence^4^, a state of irreversible cell cycle arrest, which is associated with age related pathology and phenotypic alterations^20,21^. Expression of the cyclin-dependent kinase inhibitor 2A (CDKN2A) gene is positively correlated to cellular senescence and has emerged as a valuable marker of cellular senescence over the last decade^21,22^. CDKN2A is a cell cycle inhibitor gene encoding for p16^INK4a^ and p14^arf^^20,21^. Expression of CDKN2A is positively correlated with 3-repeat TAU (microtubule-associated protein) transcripts in blood and associated with mild cognitive decline in humans^23^.

This study investigated the telomere-mitochondria axis of aging in PD patients. We investigated the association between PD with cellular aging biomarkers (telomere attrition, mitochondrial copy number) in blood. Furthermore, we investigated the association of PD, Parkinson’s Disease Dementia (PDD) and Dementia with Lewy Bodies (DLB) with cellular aging biomarkers (telomere attrition, mitochondrial dysfunction, and cellular senescence) in postmortem prefrontal cortex tissues.

## Results

### Senescence biomarkers in whole blood

**Figure 1:**
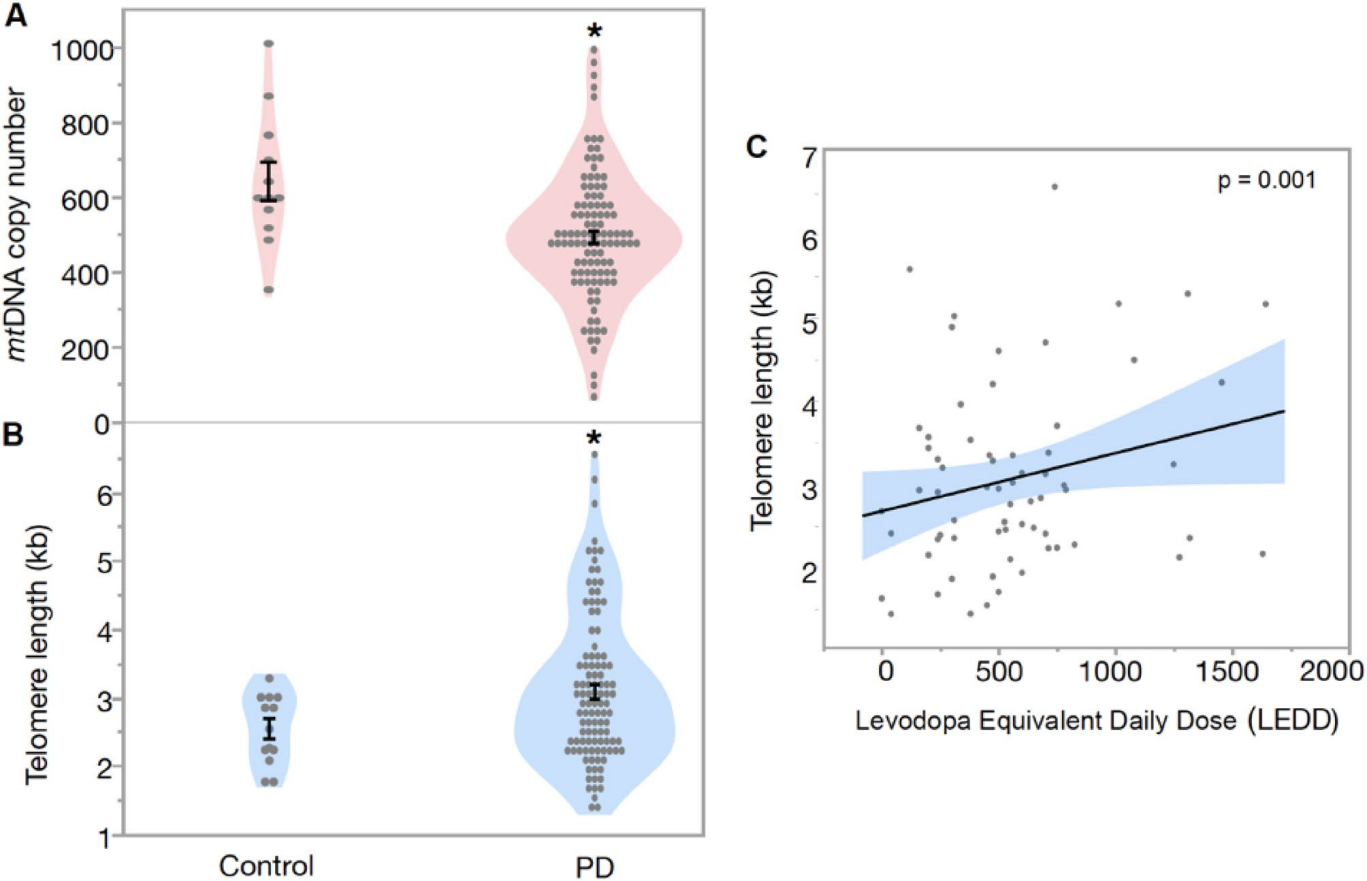
(**A**) Difference in mtDNA copy number and (**B**) telomere length between Parkinson’s Disease (PD) patients and controls in blood. Mean mtDNA copy number in PD patients was 505.99 ± 17.68, compared to controls 630.48 ± 52.88, while mean telomere length in PD patients was 3.21 ± 1.12 kb compared to the controls 2.54 ± 0.15 kb (**C**) Association between telomere length and Levodopa Equivalent Daily Dose (LEDD) in blood (multivariate regression). Data are presented as mean ± SE. *p < 0.05 versus control.

PD patients had significantly lower number of mitochondria (*Est.* = 62.74, *SE* = 27.02, *df* = 1, *t* = 2.36, *p* = 0.020, Fig. 1A,) with no significant effect of age (*Est.* =  − 1.506, *SE* = 1.764, *df* = 115, *t* =  − 0.857, *p* = 0.395) and sex (*Est.* =  − 9.237, *SE* = 16.42, *df* = 1, *t* =  − 0.56, *p* = 0.576). Our results show that PD patients had significantly longer telomeres in blood compared to controls (*Est.* =  − 0.393, *SE* = 0.176, *df* = 1, *t* =  − 2.23, *p* = 0.028, Fig. 1B), with no effect of age (Est. = 0.009, SE = 0.011, df = 115, t = 0.84, p = 0.400) and sex (*Est.* = 0.188, *SE* = 0.107, *df* = 1, *t* = 1.75,* p* = 0.083). Overall PD patients had 19.7% lower *mtDNA* copy number and 26.3% longer telomeres compared to controls (Fig. 1A,B). Mitochondrial DNA copy number and telomere length showed no significant correlation between each other in blood, neither for PD patients nor for controls (all *p* > 0.05, data not shown).

**Figure 2:**
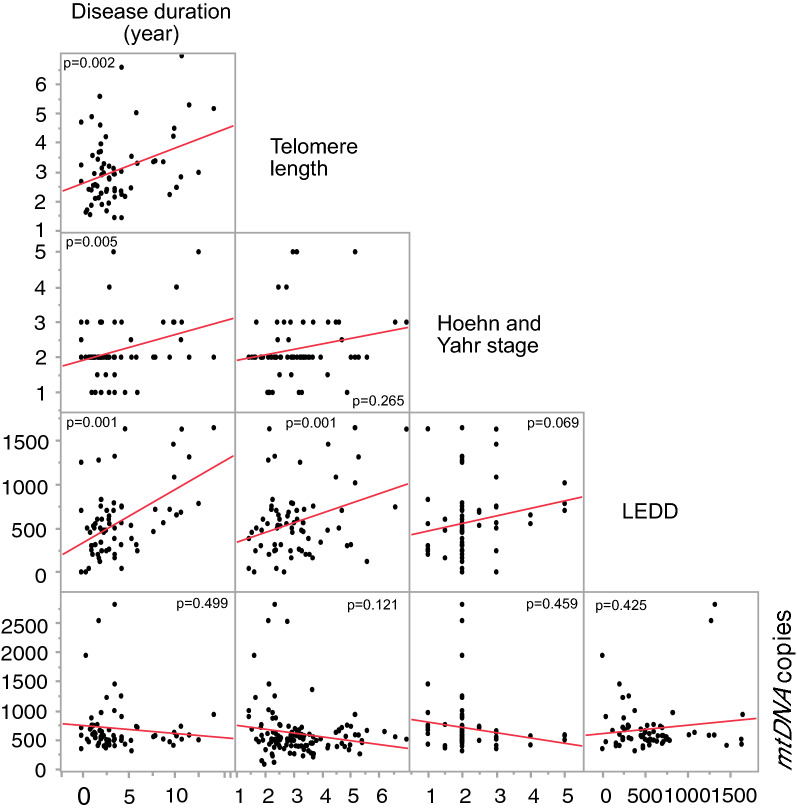
Correlation between Disease duration (number of years since the PD diagnosed), telomere length, disease severity (Hoehn and Yahr), Levodopa Equivalent Daily Dose (LEDD) and mitochondria copy number. *P* values are reported from multivariate annova, test including age and sex as co-factors in the model.

Our multivariate regression analysis showed that telomere length was positively correlated with LEDD in PD patients (*p* = 0.015, Fig. 1C) with a significant effect of age and sex (all *p* < 0.05, Table S1), while no such correlation was found for mtDNA copy number and LEDD (Table S1). Furthermore, disease duration (number of years since the PD diagonised) was positively correlated with disease severity measured by Hoehn and Yahr (*p* = 0,005, Fig. 2), telomere length (*p* = 0.002) and LEDD (*p* < 0.001) after accounting for age and sex. However, disease duration was not correlated with *mtDNA copy number* in PD patients (*p* = 0.499), when controlling for age and sex (Table S2). However, there was no correlation of mtDNA copy number and telomere length in PD with any studied clinical parameter (MDS-UPDRS III, Hoehn and Yahr, MADRS, HADS-Anxiety, HADS-Depression and MoCA; all *p* > 0.05, data Table S3).

### Senescence biomarkers in brain tissue

**Figure 3:**
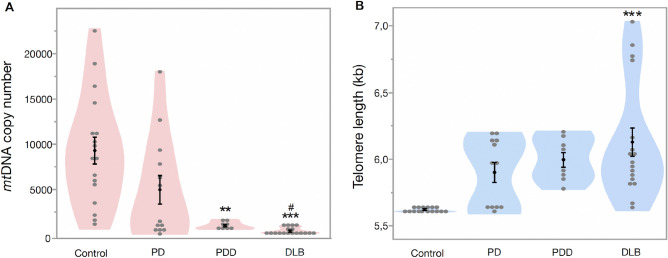
Mitochondrial DNA copy number and telomere length in prefrontal cortex tissue. (**A**) Mean MtDNA copy number in controls (9357.3 ± 1487.4), PD (5015.5 ± 1578.8), PDD (1036.4 ± 119.3) and DLB (410.7 ± 71.3). (**B**) Mean telomere length in control (5.60 ± 0.001 kb), PD (5.89 ± 0.07 kb), PDD (5.99 ± 0.01 kb) and DLB (6.12 ± 0.10 kb). Data are presented as mean ± SE. Controls vs patients, *p* < 0.05 = *, *p* < 0.005 = **, *p* < 0.0005 = *** and PD versus PDD/DLB, *p* < 0.05 = #, *p* < 0.005 = ##.

To assess whether our findings in whole blood DNA are comparable to patient’s prefrontal cortex tissue, we studied the *mtDNA* copy number and telomere length in brain tissue from patients with PD, PDD, DLB and healthy controls. Our multivariate regression model shows that patients had significantly lower mtDNA copy number in prefrontal cortex tissue, compared to controls (*Est.* = 5075.89, *SE* = 1016.04, *df* = 3, *t* = 5.00, *p* < 0.001, Fig. 3A), with no significant effect of age (*Est.* = 125.71, *SE* = 94.54, *df* = 50, *t* = 1.33, *p* = 0.189) and sex (*Est.* = 155.95, *SE* = 622.2, *df* = 1, *t* = 0.25, *p* = 0.803). Comparison of the groups was further explored using a multiple comparison test (Tukey’s test), which showed that both PDD and DLB had significantly lower mitochondrial copy number (88.9% and 95.6%, respectively) compared to controls (all *p* < 0.05, Table 1). Furthermore, *mtDNA* copy number was 91.8% lower in DLB compared to PD (*p* = 0.011, Table 1).

**Table 1:** Multi comparison (post hoc Tukey analysis) of *mtDNA* copy number, telomere length, *PGC1a*, *PGC1b* and *CDKN2A* between patient and controls in brain tissues.

*Condition*	*Condition*	*Mean diff*	*SE*	*t ratio*	*Adj. p value*	*Lower 95*	*Upper 95*
***mtDNA* copy number**							
Control	PD	3584.95	1732.17	2.07	0.177	105.79	7064.12
Control	PDD	7471.8	1980.2	3.77	**0.002**	3494.46	11,449.16
Control	DLB	9246.82	1498.03	6.17	**< 0.001**	6237.92	12,255.73
PD	PDD	3886.85	1962.35	1.98	0.208	− 54.64	7828.35
PD	DLB	5661.87	1754.23	3.23	**0.011**	2138.39	9185.35
PDD	DLB	1775.01	2012.81	0.88	0.382	− 2267.84	5817.87
**Telomere length**							
Control	PD	− 0.22	0.125	− 1.76	0.303	− 0.473	0.032
Control	PDD	− 0.322	0.136	− 2.37	0.097	− 0.596	− 0.048
Control	DLB	− 0.514	0.101	− 5.06	**< 0.001**	− 0.718	− 0.309
PD	PDD	− 0.101	0.131	− 0.77	0.866	− 0.365	0.162
PD	DLB	− 0.293	0.124	− 2.35	0.100	− 0.544	− 0.042
PDD	DLB	− 0.191	0.135	− 1.42	0.496	− 0.464	0.08
**PGC1 α**							
Control	PD	0.463	0.257	1.8	0.282	− 0.05	0.976
Control	PDD	− 0.053	0.213	− 0.25	0.994	− 0.479	0.372
Control	DLB	0.007	0.206	0.04	0.999	− 0.405	0.420
PD	PDD	− 0.516	0.226	− 2.28	0.113	− 0.969	− 0.063
PD	DLB	− 0.455	0.234	− 1.94	0.221	− 0.924	0.013
PDD	DLB	0.060	0.186	0.33	0.987	− 0.311	0.432
**PGC − 1β**							
Control	PD	0.531	0.182	2.91	**0.024**	0.166	0.895
Control	PDD	0.395	0.149	2.65	**0.048**	0.097	0.694
Control	DLB	0.355	0.147	2.41	0.085	0.060	0.649
PD	PDD	− 0.135	0.161	− 0.84	0.835	− 0.457	0.186
PD	DLB	− 0.176	0.166	− 1.06	0.717	− 0.509	0.156
PDD	DLB	− 0.040	0.130	− 0.31	0.989	− 0.300	0.219
**CDKN2A**							
Control	PD	− 0.041	0.373	− 0.11	0.999	− 0.786	0.702
Control	PDD	0.057	0.318	0.18	0.997	− 0.577	0.692
Control	DLB	− 1.012	0.301	− 3.36	**0.006**	− 1.613	− 0.411
PD	PDD	0.099	0.327	0.30	0.990	− 0.554	0.752
PD	DLB	− 0.970	0.330	− 2.94	**0.022**	− 1.630	− 0.311
PDD	DLB	− 1.069	0.269	− 3.97	**0.001**	− 1.607	− 0.532

Significant are in value [bold].

Telomere length in prefrontal cortex tissue was significantly longer in patients than controls (*Est.* =  − 0.264, *SE* = 0.071, *df* = 3, *t* =  − 3.68, *p* < 0.001, Fig. 3B) with no significant effect of age (*Est.* =  − 0.007, *SE* = 0.006, *df* = 47, *t* =  − 1.09, *p* = 0.280) and sex (*Est.* = 0.022, *SE* = 0.043, *df* = 1, *t* = 0.51, *p* = 0.611). Further group comparison (Tukey’s test) showed that the DLB group had significantly longer telomere length (9%) compared to controls (*p* < 0.05, Table 1). However, there was only a tendency of longer telomeres in the PDD group compared to controls (*p* = 0.097, Table 1).

**Figure 4:**
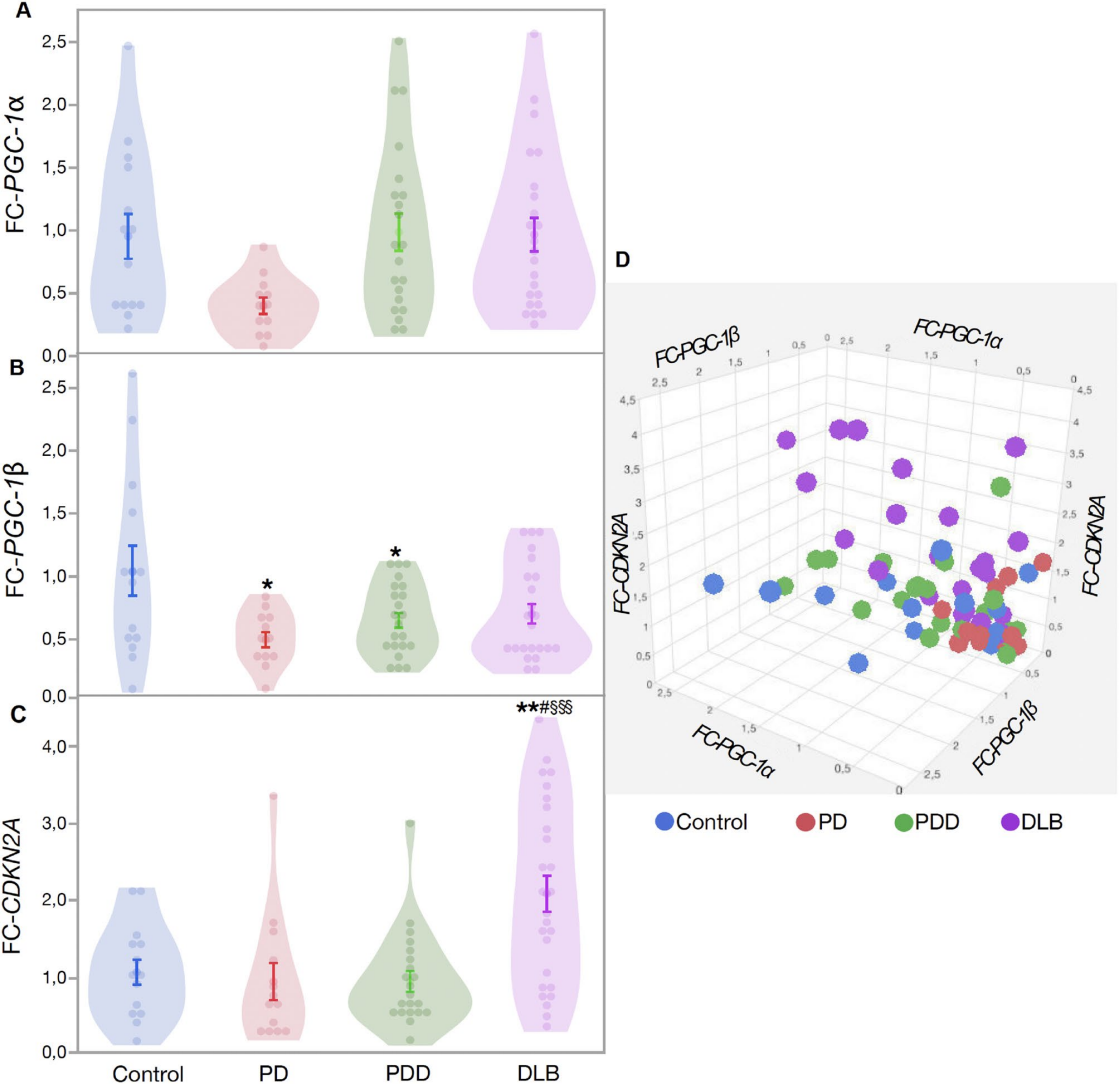
Change in PGC-1α, PGC-1β and CDKN2A gene expression in brain tissues. (**A**) Fold change of PGC-1α in patients compared to controls, (**B**) fold change of PGC-1β in patients compared to controls, (**C**) fold change of CDKN2A in patients compared to controls, (**D**) 3D graph showing the relation between expression of PGC-1α, PGC-1β and CDKN2A in patients and controls. Data are presented as mean ± SE. Controls versus patients *p* < 0.05 = *, *p* < 0.005 = **, *p* < 0.0005 = ***; PD versus PDD/DLB, *p* < 0.05 = #, *p* < 0.005 = ##, *p* < 0.0005 = ###; PDD versus DLB, *p* < 0.05 = §, *p* < 0.005 = §§, *p* < 0.0005 = §§§.

Next, we studied mitochondrial biogenesis (*PGC-1α and PGC-1β****)*** and cellular senescence (*CDKN2A*) gene expression in prefrontal cortex tissue. Our multivariate analysis did not show a significant difference between patients and controls for *PGC-1α* gene expression (*Est.* = 0.014, *SE* = 0.141, *df* = 3, *t* = 0.74, *p* = 0.464, Fig. 4A) with no significant effect of age (*Est.* = 0.012, *SE* = 0.011, *df* = 64, *t* = 1.01, *p* = 0.316) and sex (*Est.* = 0.041, *SE* = 0.082, *df* = 1, *t* = 0.50, *p* = 0.620). Further group comparison with Tukey’s test showed similar results (Table 1). *PGC-1β* expression was significantly lower in patients (*Est.* = 0.320, *SE* = 0.100, *df* = 3, *t* = 3.19, *p* = 0.002, Fig. 4B) with no significant effect of age (*Est.* = 0.002, *SE* = 0.008, *df* = 65, *t* = 0.34, *p* = 0.735) and sex (*Est.* = 0.035, *SE* = 0.057, *df* = 1, *t* = 1.62, *p* = 0.536, Fig. 3B). Further multi comparison test (Tukey) shows that controls have higher *PGC-1β* expression when compared to PD (*p* = 0.024), PDD (*p* = 0.048) and a tendency for DLB group (*p* = 0.085), (Table 1).

Overall, *CDKN2A* expression was not significantly higher in patients compared to controls (*Est.* =  − 0.249, *SE* = 0.209, *df* = 3, *t* = -1.19, *p* = 0.239, Fig. 4C) with no significant effect of age (*Est.* = 0.023, *SE* = 0.017, *df* = 68, *t* = 1.35, *p* = 0.180) and sex (*Est.* =  − 0.008, *SE* = 0.121, *df* = 1, *t* =  − 0.07, *p* = 0.944). When using multiple comparison test (Tukey), the DLB group shows significantly higher *CDKN2A* expression compared to controls, PD and PDD (all *p* < 0.05, Fig. 4C, Table 1). Our results show that patient groups (PD, PDD, and DLB) had significantly lower *mtDNA* copy number and *mtDNA* biogenesis gene expression levels, but higher cellular senescence gene expression, with DLB showing the strongest effect among all patient groups (Fig. 4D). To further investigate the correlation between different variables, we pooled all the patient data to increase our sample size. In patients, telomere length was negatively correlated with mitochondrial copy number (*r*^*2*^ = 0.167, *N* = 36, *p* = 0.012) and positively correlated with *CDKN2A* expression (*r*^*2*^ = 0.286, *N* = 18, *p* = 0.018), while *PGC-1α* and *PGC-1β* were positively correlated with each other (*r*^*2*^ = 0.543, *N* = 55, *p* < 0.001).

## Discussion

Using blood and prefrontal cortex brain tissues from two different cohorts we show that mitochondrial dysfunction (*mtDNA* copy number and *mitochondria* biogenesis gene expression) and cellular senescence, but not telomere shortening is associated with neurodegenerative diseases (PD, PDD and DLB). Our results suggest that mitochondrial dysfunction (copy number and biogenesis) in blood might be a valuable marker to assess the early risk of PD.

A single mitochondrion contains 2–10 copies of *mtDNA*, depending on the type of cell and tissue^24^. Under healthy circumstances, human cells contain thousands of copies of mtDNA that are usually identical (homoplasmy). However, during infection or disease settings, mtDNA frequently presents a mixture of wild-type mtDNA within each cell^25^, therefore, mutant mitochondrial genome accumulates in cells over time^4^. The number of *mtDNA* copies increases with age, as a compensatory mechanism, which maintains the amount of wild-type *mtDNA* and reverses the effect of defective mitochondria accumulation^26^. However, this compensatory mechanism declines in PD resulting in exhaustion of *mtDNA copies*, which, in turn, leads to respiratory deficiency in dopaminergic neurons^26^. Here, we report a significant reduction of *mtDNA* copy number in both blood and prefrontal cortex brain tissues of PD, PDD and DLB patients, compared to healthy controls (Figs. 1A, 2A). In accordance, previous studies have shown that PD patients have lower *mtDNA* copy number in blood compared to healthy controls^27–29^. We found similar mitochondrial reduction (20%) in whole blood in PD patients compared to 19.6% in PBMC previously reported by Pyle et al.^28^. However, we found lower mtDNA copy numbers (46.4%) in prefrontal cortex tissues, while Pyle et al. showed no significant difference of mtDNA copy numbers between PD patients and controls in frontal cortex^28^. Overall, our results are also in agreement with findings from other neurodegenerative diseases including Alzheimer’s disease (AD) and Huntington’s disease, where mitochondrial dysfunction is observed^28,30,31^.

Mitochondrial copy number is strongly associated with mitochondrial function, which makes it an important aging marker^32^. *MtDNA* mutation and mitochondrial dysfunction, respectively, have been associated with neurodegenerative diseases such as PD and AD^5,33,34^. Our study also shows lower expression of *PGC-1β* gene (master regulators of mitochondrial biogenesis) in brain tissues of PD, PDD and DLB patients compared to healthy controls (Fig. 4B), However, the expression level of *PGC-1α* was similar between patients and controls. A recent study by Dölle et al. 2016 also showed no difference in *PGC-1α* between PD patients and controls^35^. Lack of of *PGC-1α* correlation could be explained due to the fact that *PGC-1α* also influences the expression of several other genes involved in metabolic pathways^36^, and therefore its expression might be highly regulated to avoid its deleterious side effects. Our study suggests that both lower *mtDNA* copy number and expression of *PGC-1β* in PD, PDD and DLB might indicate dysfunctional mitochondria in patients. An alternative explanation of low mitochondrial content could be due the presence a of high proportion of mutant mitochondrial genome. Future studies characterizing the mitochondrial genome into mutant and wild-type can further explain the mitochondrial role in neurodegenerative diseases.

Our results show that PD patients have longer telomeres in blood compared to healthy controls (Fig. 1B). We found similar results in brain tissues where PD, PDD and DLB patients show longer telomeres compared to healthy controls (Fig. 3B). These results were contrary to our expectations as we expected that patients would show shorter telomeres compared to controls. So far, previous literature has shown mixed results, no association of telomere length with PD in blood^37–41^ and brain tissue^42^. However, a study by Maeda et al., 2012, from Japanese women reported shorter blood telomere length in PD patients^43^. Similarly, DLB patients have been shown to have shorter telomeres compared to controls^44^. However, a recent nested case control study showed a positive association between PD and longer telomere length in leukocytes and PBMCs, where men with shorter telomere length were of lower risk of getting diagnosed with PD^45^. Furthermore, Degerman et al. 2014 reported that PD patients who developed dementia within three years after diagnosis had longer telomere length at diagnosis compared to the other PD patients without early development of dementia^37^.

Contradictory results of telomere association with PD could be due to the heterogeneity of the study setup (cross sectional vs nested case control), sample heterogeneity, quality and cell composition within each tissue, or differential methods for assessing telomere length. An alternative explanation could be the effect of PD medication on telomere length. Interestingly, our results show that blood telomere length was significantly positively correlated with Levodopa Equivalent Daily Dose (LEDD) medication in PD patients. Furthermore, we found a positive correlation of telomere length with disease duration (years since PD diagnosis) in PD patients, which may further reflect the cumulative effect of LEDD on telomere length. Previously it has been shown that Levodopa moderately induces nerve growth factor and growth hormone^46^. In addition, Levodopa increased homocysteine, which in turn may accelerate aging processes, such as neuropathy and dementia^47^. Our results show that disease duration is positively correlated with Levodopa Equivalent Daily Dose LEDD, which in turn positively correlated with telomere length in blood in PD patients. Our results show that DLB patients have longer telomeres in brain tissue than controls and other patient groups. We did not have Levodopa Equivalent Daily Dose (LEDD) nor other clinical information for the brain tissues as we did for the blood samples. We were, therefore, unable to investigate whether this was an effect of the medication, as generally DLB patients are treated with lower LEDD than PD patients. Our study is also limited in terms of varying cell composition within each brain tissue which might also affect our results. Nevertheless, to elucidate the relationship between neurodegenerative diseases and telomere length, and to pinpoint whether short/long telomeres are the cause or consequence of neurodegenerative diseases, a longitudinal study set-up is needed, with well-defined samples.

Here, we show a significantly higher expression of cyclin dependent kinase inhibitor 2A (CDKN2A) in prefrontal cortex brain tissue of DLB patients compared to healthy controls. CDKN2A reflects the increased load of cellular senescence and has been shown to be negatively associated with telomere length^15,21,22^. A previous study showed that expression of CDKN2A has been associated with mild cognitive decline in aging humans, where CDKN2A expression was positively associated with 3 repeat TAU (microtubule-associated protein) in blood^23^. However, contrary to previous findings we found a positive correlation between telomere length and CDKN2A expression in PD patients. Mechanisms behind such association are yet to be investigated. One strength of the study is that different tissues were compared (blood vs brain) in Parkinson’s disease (PD). However, it is difficult to obtain large sample sizes when using brain tissue and therefore results should be taken with cautions.

Our results show that mitochondrial DNA copy number and telomere length are not correlated in blood. However, in a previous study with healthy individuals we showed that these two markers were positively correlated^48^. Telomere-mitochondria axis is argued to compromise metabolism and organ function, where telomere dysfunction triggers P53 and P16, which in turn affect mitochondrial biogenesis^49^. We show a negative correlation of CDKN2A (P16) expression with mitochondrial copy number in brain tissues. Lack of correlation between telomere length and mitochondria in brain tissues could depend upon frequent mtDNA mutation due to disease, which accumulate over the time^25^. Hence our primers only capture wild type mitochondria and not all mitochondrial DNA.

In conclusion, our results indicate that mitochondrial dysfunction and cellular senescence might be valuable markers to study neurodegenerative diseases (PD, PDD, DLB). Follow-up studies were more individuals, particularly healthy controls, are important to perform. The identification of blood biomarkers in neurodegenerative diseases could potentially facilitate the drug development process, as the utility of measuring such markers in the brain is limited. Our findings further extend our knowledge that mitochondrial copy number and function could be a viable biomarker in blood as an early indicator for the risk of neurodegenerative diseases.

## Materials and methods

### Blood samples from Swedish cohort

The blood samples were obtained from controls and PD patients from the Swedish BIOPARK cohort, following the Declaration of Helsinki and Good Clinical Practice standards and approved by the Swedish Ethical Review Authority, reference number 2019–04967 and all applied methods were carried out in accordance with relevant guidelines and regulations^50^. Patients were recruited in clinics within Stockholm region, Sweden, and from the Sunderby Hospital in Luleå, Sweden. Both verbal and written informed consent were obtained at the time of inclusion. Blood was drawn by venepuncture by trained personnel and collected in EDTA tubes. The *mt*DNA copy number and the telomere length were measured from DNA of whole blood of *n* = 112 individuals including 100 PD patients and 12 controls. Age range of patients diagnosed with PD was between 47–97 years and male/female ratio was 1.5, while controls had an age range between 54–73 and male/female ratio was 0.3. Clinical data was collected from PD patients including, disease duration (years since PD diagnosis), Movement Disorder Society Unified Parkinson’s Disease Rating Scale part 3 (MDS-UPDRS III) for motor symptoms, Hoehn and Yahr for disease severity, Montgomery-Åsberg Depression Rating Scale (MADRS) for depression, Hospital Anxiety and Depression Scale sub scores for anxiety (HADS-Anxiety) and depression (HADS-Depression), Montreal Cognitive assessment (MoCA) for cognitive assessment, and Levodopa Equivalent Daily Dose (LEDD) as a standard measure for patients’ dopaminergic medication.

### Brain tissues samples from UK cohort

Postmortem human prefrontal cortex brain tissues were obtained from the MRC London Neurodegenerative Diseases Brain Bank, King’s College London, United Kingdom. The permission to collect human brain tissue included participants informed consent for research purposes following the procedure corresponds to principles expressed in the Declaration of Helsinki and Good Clinical Practice standards and approved by the UK National Research Ethics Service (08/H1010/4 and KI IRB)^51^. All applied methods were carried out in accordance with relevant guidelines and regulations. In total 58 brain tissues were used including 13 PD patients, 8 PDD patients, 19 DLB patients and 16 healthy controls (for demographic characteristics of donors, see Table 2).

**Table 2:** Demographic characteristics of UK brain tissues samples.

	*Number*	*Age range (years)*	*M/F ratio*
**DNA**			
Control	16	68–96	1.6
Parkinson’s disease PD	13	69–89	1.1
Parkinson’s disease dementia PDD	8	68–81	1.6
Dementia with Lewy bodies DLB	19	74–92	0.9
**RNA**			
Control	13	66–96	1.6
Parkinson’s disease PD	13	59–89	1.1
Parkinson’s disease dementia PDD	22	68–89	1.2
Dementia with Lewy bodies DLB	27	65–92	1.1

### Telomere and mitochondrial copy number assay

DNA was extracted using QIAmp DNA Blood Maxi Kit (Cat# 51,994, QIAGEN) according to manufacturer’s instructions. DNA concentration was measured using a Nanodrop (Marshall Scientific). In brain tissues DNA was extracted using 30 mg frozen human brain tissue using QIAmp DNA Blood Maxi Kit. DNA concentration and purity was measured using a Nanodrop (Marshall Scientific). Telomere length and mitochondrial copy number was measured using the ScinceCell kit (cat# 8958) from blood and brain tissues DNA. Each 15 μl reaction contained 7.5 μl QuantiNova Syber green (cat # 208,054, Qiagen), 0.5 μl telomere or single copy (SCR) or mitochondria primers, 0.1 μl ROX (passive reference dye), 1.9 μl DNA/RNA free water and 5 μl (1 ng/μl) template DNA. For telomere qPCR, the thermal cycle profile included incubation at 50 °C for 2 min and 95 °C for 10 min before running 30 thermal cycles (95 °C for 15 s, 56 °C for 45 s, and 72 °C for 45 s). For single‐copy gene and mitochondrial copy number qPCR, the thermal profile included incubation at 50 °C for 2 min and 95 °C for 10 min before running 40 thermal cycles (95 °C for 15 s, 54 °C for 45 s, and 72 °C for 45 s). Each assay was run on a separate plate, with each plate containing a serially diluted DNA sample to calculate the PCR efficiency. PCR acceptance value was set to 100 ± 15%, any plate producing the PCR efficiency outside this range was repeated. Samples were run in triplicate, and mean *C*_*T*_ value was used for final calculation after carefully checking the melt curve for each sample.

A reference genomic human DNA of known telomere length of 4.1 kb (or 369 kb, per diploid cell) and mitochondrial copy number (1200 ± 9 copies) was added on each plate. Δ*C*_*T*_ for both telomere length and mitochondrial copy number was calculated using the formula *C*_*T*_ target sample–*C*_*T*_ reference sample after adjusting the PCR efficiency using Pfaffl method^52^. We then calculated the ∆∆*C*_*T*_ for both telomere length and mitochondrial copy number using the formula (TEL∆*C*_*T*_–SCR∆*C*_*T*_). Relative telomere length of target sample to reference sample was calculated as 2^ − ∆∆*C*_*T*_ and the ratio was then multiplied with 369 kb to get telomere length per diploid cell. Telomere length of the diploid cell was divided by number of chromosomes ends (92) to get average telomere length of each chromosome end (2^ − ΔΔ*C*_*T*_ × 369/92). Mitochondria copy number per diploid cell of target sample to reference sample was calculated as 2^ − ∆∆*C*_*T*_ and the ratio was then multiplied with 1200 mtDNA copy number for each sample (2^ − ΔΔ*C*_*T*_ × 1200), as described elsewhere^48^.

### Gene expression

30 mg frozen human brain tissue was used to extract RNA using RNeasy Plus Mini Kit (Qiagen) according to manufacturer’s protocol. RNA concentration was measured and evaluated for purity (260/280 nm ratio) using Nanodrop (Marshall Scientific). RNA integrity was confirmed using bioanalyzer. cDNA was synthesized by using QuantiTec Reverse Transcriptase kit (cat# 205,311) following the manufacturer guidelines. Thermal profile consisted of 10 min incubation at 25 °C, followed by 1 h at 42 °C for cDNA synthesis and 5 min at 85 °C to inactivate the enzyme on a Quant Studio 5 thermocycler. Relative gene expression of *CDKN2A*, *PGC1 α* and *PGC-1β* was determined using the comparative ∆*C*_*T*_ method by calculating the *C*_*T*_ values of the target genes (*CDKN2a*, *PGC1 α* and *PGC-1β*) against the *C*_*T*_ values of the reference gene (*GAPDH*). Target genes and *GAPDH* were run in triplicates and amplified in the same wells. Respective *C*_*T*_ values were averaged before performing the ∆*C*_*T*_ calculation (∆*C*_*T*_ = *C*_*T*__Target_ − *C*_*T*__GAPDH_). Gene expression values were converted into log 2 of ∆*C*_*T*_ (2^ − ∆*C*_*T*_).

### Cellular senescence and mitochondrial biogenesis

*CDKN2A, PGC1α* and *PGC-1β* expression was measured using TaqMan Gene Expression Assay (*CDKN2A,* HS00923894_m1; *PGC1α,* Hs00173304_m1; *PGC-1β,* Hs00993805_m1; Applied Biosystem) on a Quant Studio 5 qPCR instrument. The total qPCR reaction of 20 μl contained 3 μl cDNA, 10 μl TaqMan Multiplex Master Mix (cat # 4,461,882; Applied Biosystem), 1 µl GAPDH Assay (cat # 4,485,712; Applied Biosystem), 1 μl of *CDKN2A*, *PGC1α* and *PGC-1β* Assay and ddH2O. TaqMan *GAPDH* Assay was added to each run as an endogenous control. Thermal profile included 95 °C for 20 s, followed by 45 thermal cycles (95 °C for 1 s and 60 °C for 20 s).

### Statistical analysis

Statistical analysis was performed using the statistical program JMP (version 16). We performed multivariate regression analysis to investigate the correlation of disease with three hallmarks of aging (telomere attrition, mitochondrial dysfunction, and cellular senescence) in blood and brain tissue separately. Age and sex were fitted as fixed factors in all analysis. For further comparison between different groups, we used multiple comparison test (Tukey’s test). Pearson correlation was used to assess the correlation between different cellular aging markers. Fold change of *PGC1α, PGC-1β* and *CDKN2A* was calculated by dividing the individual values with the mean value of controls.

### Institutional review board statement

The blood samples were obtained from PD patients included in the Swedish BIOPARK cohort (approved by the Swedish Ethical Review Authority, reference number 2019–04967)^50^. Patients were recruited in clinics within Stockholm region, Sweden, and from the Sunderby Hospital in Luleå, Sweden. Both verbal and written consent were obtained at the time of inclusion. Postmortem human prefrontal cortex brain tissues were obtained from the MRC London Neurodegenerative Diseases Brain Bank, King’s College London, United Kingdom. The permission to collect human brain tissue included participants consent for research purposes and ethical approval was obtained from the UK National Research Ethics Service (08/H1010/4 and KI IRB)^51^.

### Informed consent statement

Both verbal and written informed consent were obtained at the time of inclusion.

## Supplementary Information

Supplementary Information.

## Data Availability

All data are available upon reasonable request to corresponding author, Muhammad Asghar (asghar.muhammad@biol.lu.se).
