# Supplementary material for: Mitochondrial biogenesis, telomere length and cellular senescence in Parkinson’s disease and Lewy body dementia
Source: Sci Rep. 2022 Oct 20;12:17578. doi: 10.1038/s41598-022-22400-z (PMC9584960; doi:10.1038/s41598-022-22400-z)
Supplement: Supplementary file 1 — Supplementary Information. [file 41598_2022_22400_MOESM1_ESM.pdf]

Supplementary Material

Table S1. Association between *mtDNA copy number,* telomere length and LEDD in blood in Parkinson patients (multivariate Anova).

	Est.	*SE*	*df*	*t ratio*	*p*
mtDNA copy number					
Sex	-23.61	18.91	1	-1.25	0.216
Age	1.094	1.925	63	0.57	0.572
LEDD	-0.001	0.046	63	-0.12	0.905
Telomere length					
Sex	0.332	0.135	1	2.64	**0.016**
Age	0.032	0.013	63	2.35	**0.020**
LEDD	0.001	0.0003	63	3.45	**0.001**

Table S2. Association between Disease duration, disease severity, telomere length, LEDD and *mtDNA* copy number in blood in Parkinson's patients (multivariate Anova).

	Est.	*SE*	*df*	*t ratio*	*p*
Disease severity					
Sex	0.254	0.096	1	2.63	**0.010**
Age	0.003	0.010	62	0.30	0.764
Disease duration	0.084	0.029	62	2.09	**0.005**
Telomere length					
Sex	0.370	0.136	1	2.72	**0.008**
Age	0.018	0.014	62	1.27	0.210
Disease duration	0.127	0.041	62	3.11	**0.002**
LEDD					
Sex	0.332	0.135	1	2.64	**0.016**
Age	0.032	0.013	62	-2.06	**0.020**
Disease duration	0.001	0.0003	62	3.45	**0.001**
mtDNA copy number					
Sex	-116.92	54.07	1	2.16	**0.034**
Age	-11.66	5.65	62	-2.06	**0.043**
Disease duration	-11.05	16.27	62	-0.68	0.499

Table S3. Association between *mtDNA copy number,* telomere length with clinical parameters (Hoehn and Yahr, MDS-UPDRS III, MADRS, HADS-Anxiety, HADS-Depression and MoCA).

	Est.	*SE*	*df*	*t ratio*	*p*
mtDNA copy number					
Hoehn and Yahr					
Sex	-101,5	53,47	1	-1,90	0,062
Age	-12,14	5,465	63	-2,22	**0,029**
Hoehn and Yahr	-44,96	60,41	63	-0,74	0,459
MDS-UPDRS III					
Sex	-80,47	52,15	1	-1,54	0,130
Age	-0,038	6,505	41	-0,01	0,995
MDS-UPDRS III	-1,640	5,047	41	-0,32	0,746
MADRS					
Sex	-102,1	52,44	1	-1,95	0,055
Age	-16,88	6,184	66	-2,73	**0,008**
MADRS	-2,950	6,022	66	-0,49	0,625
HADS-Anxiety					
Sex	-113,7	52,38	1	-2,17	**0,033**
Age	-15,96	5,987	66	-2,67	**0,009**
HADS-Anxiety	0,190	11,63	66	0,02	0,987
HADS-Depression					
Sex	-108,1	52,92	1	-2,04	**0,045**
Age	-16,74	6,106	65	-2,74	**0,007**
HADS-Depression	-2,609	11,09	65	-0,24	0,814
MoCA					
Sex	-92,01	50,22	1	-1,83	0,071
Age	-12,59	5,825	67	-2,16	**0,034**
MoCA	4,199	9,592	67	0,44	0,662
Telomere length					
Hoehn and Yahr					
Sex	0,237	0,147	1	1,61	0,112
Age	0,029	0,015	63	1,96	0,054
Hoehn and Yahr	0,187	0,166	63	1,12	0,265
MDS-UPDRS III					
Sex	0,412	0,141	1	2,92	**0,005**
Age	0,023	0,017	41	1,33	0,192
MDS-UPDRS III	0,010	0,013	41	0,73	0,469
MADRS					
Sex	0,287	0,150	1	1,92	0,059
Age	0,035	0,017	66	2,01	**0,048**
MADRS	0,016	0,017	66	0,93	0,354
HADS-Anxiety					
Sex	0,313	0,146	1	2,14	**0,035**
Age	0,034	0,016	66	2,09	**0,040**
HADS-Anxiety	0,017	0,032	66	0,52	0,601
HADS-Depression					
Sex	-0,326	0,142	1	2,29	**0,025**
Age	0,040	0,016	65	2,48	**0,015**
HADS-Depression	0,069	0,029	65	2,33	**0,022**
MoCA					
Sex	0,273	0,137	1	1,99	0,051
Age	0,027	0,015	67	1,75	0,085
MoCA	-0,033	0,0262	63	-1,29	0,202
